# Labor force participation in later life: Evidence from a cross-sectional study in Thailand

**DOI:** 10.1186/1471-2318-11-15

**Published:** 2011-04-08

**Authors:** Ramesh Adhikari, Kusol Soonthorndhada, Fariha Haseen

**Affiliations:** 1 Geography and Population Department, Mahendra Ratna Campus, Tribhuvan University, Kathmandu, Nepal; 2 Institute for Population and Social Research, Mahidol University, Salaya, Phutthamonthon, Nakhon Pathom 73170, Thailand; 3 Health System and Infectious Diseases Division, ICDDR, B, Dhaka, Bangladesh

## Abstract

**Background:**

The labor force participation rate is an important indicator of the state of the labor market and a major input into the economy's potential for creating goods and services. The objectives of this paper are to examine the prevalence of labor force participation among older people in Thailand and to investigate the factors affecting this participation.

**Methods:**

The data for this study were drawn from the '2007 Survey of Older Persons' in Thailand. Bivariate analysis was used to identify the factors associated with labor force participation. The variables were further examined using multivariate analysis in order to identify the significant predictors of the likelihood of older people participating in the labor force, after controlling for other variables.

**Results:**

Overall, 30,427 elderly people aged 60 or above were interviewed. More than a third (35%) of all respondents had participated in the labor force during the seven days preceding the survey. Respondents who were female (OR = 0.56), those who were older (OR = 0.47 for 70-79 and 0.21 for 80+ years), those who were widowed/divorced (OR = 0.85), those who were living with their children (OR = 0.69), those whose family income was relatively low, and those who worked in government sectors (OR = 0.33) were less likely to participate in the labor force than were their counterparts. On the other hand, those who lived in urban areas (OR = 1.2), those who had a low level of education (OR, secondary level 1.8, primary 2.4, and no schooling 2.5), those who were the head of the household (OR = 1.9), and those who were in debt (OR = 2.3) were more likely be involved in the labor force than their comparison groups. Furthermore, respondents who experienced greater difficulty in daily living, those who suffered from more chronic diseases, and those who assessed their health as poor were less likely to participate in the labor force than their counterparts.

**Conclusion:**

Labor force participation in their advanced years is not uncommon among the Thai elderly. The results suggest that improving the health status of the elderly is necessary in order to encourage their employment. By doing so, the country can fulfill the labor shortage and further improve the economic condition of the nation. The results of this study also suggest that for policies encouraging employment among older persons to succeed, special focus on the rural elderly is necessary.

## Background

The labor force participation rate is an important indicator of the state of the labor market and a major input into an economy's potential for creating goods and services [1]. But since low fertility and low mortality produce population ageing, these combined effects over time change the age structure of both the population and the labor force toward higher age groups [2]. Thailand's fertility rate decreased to below replacement level in the mid-90s. At the end of the twentieth century, Thailand experienced a transition toward population ageing as a major consequence of low fertility and increased longevity [3]. Both in absolute numbers and proportionately, the population of those aged 60 years and over has increased faster than that of the overall population. The absolute number of elderly increased from 2.4 million in 1980 to 5.8 million in 2000 and is projected to reach almost 11 million in 2020 [4]. In the developed countries, the shift in the proportion of ageing population from 7% to 14% took more than half a century while in Thailand this has occurred within only 22 years, even faster than in Japan [5].

In Thailand, nearly three-fourths (72%) of the working-age population (persons aged 15 years and older) was in the labor force in 2007 [6]. The high labor force participation rate for those aged 50-59 years (about 80% in 2007) suggests that a large number of people continue working until they are 60 years old, which is the legally set retirement age in the public sector. However, the proportion of older persons (aged 60 or above) participating in the labor force increased from 34 percent in 2003 to 35 percent in 2007 [7,8]. In terms of gender, labor force participation of women was less than it was for men, by about 10 to 15 percent for the total working-age population. This gender difference has also been observed among the elderly (male 48%; female 27%) [8].

In Thailand, the number of people in the labor force who are of entry age (15-24) is declining. This also indicates that Thailand will soon face problems of labor shortages [3]. Because of increasing ageing populations, governments in most developed countries are seeking to increase the proportion of the population still working by rising the age of retirement [9]. Given the possibility of a labor shortage if fertility continues to decline, Thailand will face new challenges and priorities in population policy. It may be assumed from the experience of the developed countries that the problems of decreased population size, population ageing, and labor shortage will affect sustainable development [3]. Developed countries such as the US have been experiencing increased rates of older workers in their labor market since 1980 [1]. Given this scenario, Maton et al have suggested that productivity could be increased by improving the health of skilled, experienced older people by improvements in biotechnology, nanotechnology, and medicine [10].

Factors contributing to labor force participation among older people vary as a result of particular economic conditions and stages of development in different economies. Clark and Anker have estimated the effect of several indicators of economic development including per capita income on the participation rate (in 1980) of older people in 102 countries [11,12]. They found a negative relationship between income and labor force participation rates. Raymo and Cornan suggest that social security, private pensions, changes in occupational structures among older people, and economic growth of the country affect whether older people continue to work [13]. On the other hand, Alavinia and Burdorf argue that in most European countries, poor health, chronic disease, lower levels of education, and, for women, being a homemaker are the major factors associated with withdrawal from the labor force [14]. In developing countries, access to employment, public services and programs, and household and social networks are the key deciding factors as to whether older people continue working. Households and communities, especially, play a significant role as a major source of support for the elderly [15]. In South Korea, educational level and family economic status have been identified as determining factors in labor market participation [16].

In light of rapidly changing demographic events and economic growth, the labor market of Thailand has experienced changes in the composition, structure, and occupational patterns of the labor force. But it is not yet clear just which factors drive older people to continue or to stop working in the present Thai context. The objectives of this paper are to examine the prevalence of labor force participation and to investigate the factors affecting labor force participation among the elderly in Thailand. It thus aims to address the gap in knowledge by providing information on labor force participation that could assist program managers of GOs/(I)NGOs and Thai government policy makers in understanding the various factors influencing labor force participation and in designing appropriate interventions focusing on elderly people.

## Methods

### Study design and data collection

The data for this study were drawn from the '2007 Survey of Older Persons' in Thailand, a nationally representative survey conducted by the National Statistics Office (NSO). The NSO has conducted three nationally representative household surveys of older persons-in 1994, 2002, and 2007-to fulfill the need for adequate information in order to develop appropriate policies and programs to ensure the well-being of the Thai elderly [7,8]. These surveys collected information on socioeconomic conditions and living arrangements, employment and income, health status and health behavior, etc. of the elderly in Thai society.

A stratified two-stage sampling procedure was employed to collect the information. The primary sampling units were blocks for municipal areas and villages for non-municipal areas. The secondary sampling units were households, selected via random sampling from the list of all enumerated households in each block or village of the first sampling. Fifteen households per block in municipal areas and 12 households in non-municipal areas were selected as sample households. The survey was based on a national probability sample of persons aged 50 years old and older in private households who were usual residents of the household. In total, 56,002 persons were interviewed. A structured questionnaire was used. Interviews were conducted at the household level from the selected subjects by a trained interviewer. This paper analyzes the information from people aged 60 years and above. This study was approved by the ethics committee of the NSO of Thailand.

### Variables

#### Dependent variable

##### Labor force participation

Information about labor force participation was collected among older people in the 7 days preceding the survey.

#### Independent variables

*Demographic variables *included age, sex, place of residence, and marital status. *Economic variables *included average total income per year, whether or not one worked in a government job, whether or not the respondent had someone to assist him or her incase of financial problems, and current debt. *Social variables *were level of education, household headship, number of children, and whether the respondent was living with his or her children. *Health behavior variables *included functional status, number of chronic diseases, number of psychosocial symptoms, and self-assessed health status.

##### Chronic diseases

Individuals were asked about the presence of hypertension, heart disease, diabetes, cancer, stroke, and paralysis. One composite indicator-"chronic diseases condition" was constructed. Then it was categorized into four groups: (1) had no chronic disease, (2) had 1 chronic disease, (3) had 2 chronic diseases, and (4) had 3 or more chronic diseases.

##### Functional status

To measure functional status respondents were asked, "Can you perform these (.....) activities by your own?" Five activities of daily living (ADLs) (eating, getting dressed, bathing, going to the toilet, and sitting) and five instrumental activities of daily living (IADLs) (carrying things weighing 5 kgs, walking 200-300 meter, walking up 2-3 flights of stairs, taking a bus alone, and being able to calculate and use money correctly) were included in the question. A composite index of "functional status" was made from the above-mentioned questions. Then the variable was categorized into three categories: able to do all ADLs, difficulty in 1 or 2 ADLs, and difficulty in 3 or more ADLs.

##### Psychosocial symptoms

For psychosocial symptoms respondents were asked, "How often did you experience the following symptoms during the previous month?" Seven prompted symptoms were presented to the respondent, namely: (1) stress, (2) unhappiness, (3) moodiness, (4) hopelessness, (5) uselessness, (6) lack of appetite, and (7) loneliness. One composite index, "psychosocial symptoms" was created and divided into four groups: no symptoms, 1-2 symptoms, 3-4 symptoms, and more than 5 symptoms.

##### Self-assessed health

Five response categories were used to collect information on the reported self-assessed health status of the elderly. We categorized the variable into two groups: 'good health' which included 'very good' and 'good'; and 'poor health' which included 'fair', 'bad', and 'very bad'.

### Methods of analysis

The analysis is confined to those who are aged 60 years or more. Univariate, bivariate, and multivariate analysis were performed to analyze the data. Initially, univariate or descriptive analysis was used to describe the percentage of the respondents' sociodemographic characteristics. Bivariate analysis was performed to identify the factors associated with labor force participation in the week preceding the survey. A chi-square test was used to test the association between the variables. The variables were further examined in the multivariate analysis (logistic regression) in order to identify the significant predictors of the likelihood of participating in the labor force after controlling for other variables. During the process of analysis, multi-collinearity among the variables was assessed. None of the variables were highly correlated (r >0.6), so all the variables were included in the logistic model.

## Results

More than half of the sampled respondents (53%) were aged 60-69 years while about one out of seven (13%) were aged 80 years or above. Nearly three out of five respondents (57%) were female. Similarly, a large majority of the respondents (72%) had only a primary/elementary level education. About three in five respondents (58%) resided in rural areas. A notable proportion (37%) was widowed/divorced. More than half the respondents (55%) were heads of households. Similarly, more than a half (51%) reported that their family's average total income per year was 30,000 baht or less. It is also notable that about one in ten elderly (9%) was in debt during the survey period. About two in five respondents (38%) reported that they had difficulty with at least one activity in daily living, and more than two in five respondents (44%) reported that they had at least one chronic disease. More than two-thirds (69%) of respondents reported that they had at least one symptom of psychosocial problems (data not shown).

**Figure 1:**
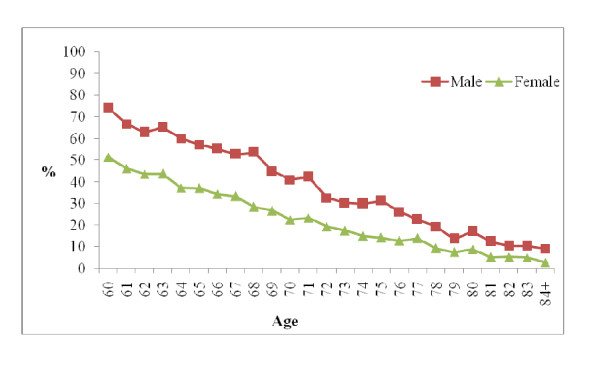
**Labor force participation by age and sex of the respondents**.

Overall, more than a third (35%) of the elderly had participated in the labor force during the seven days preceding the survey. Stratifying the respondents by age and sex shows that the percentage of respondents who had worked in the 7 days preceding the survey varied largely according to age and sex. Our study found that as age increased, labor force participation decreased. We can clearly see in Figure 1 that about three-fourths of male elderly (74%) while only about half of female elderly (51%) at age 60 were working. This percentage decreased for both sexes and fell to about three-fifths for males (59%) and only about one-fifth for females (22%) at age 70. Percentages further decreased to 17 per cent for males and 9 per cent participation for females by age 80. It is notable that only about a tenth of males (9%) and very few females (3%) aged 84 or above were working in the 7 days preceding the survey (Figure 1).

Labor force participation also varied greatly with different socio-economic and health characteristics. For instance, a significantly higher proportion of respondents who resided in urban areas, who were married, who were heads of households, who were not living with their children, who had never worked for the government or a government enterprise, who were in debt were more likely to be part of the labor force than were their counterparts. Furthermore, the percentage of those participating in the labor force decreased among those who had relatively more chronic disease, and who suffered from psychosocial symptoms. Finally, a lower percentages of respondents who assessed their health as poor were in the labor force compared to those who assessed their health as good (Table 1).

**Table 1:** Characteristics of the elderly according to labor force participation

Characteristics	Working in the past 7 days			
	
	Yes	No	Total	N
Place of residence ***				
Rural	32.0	68.0	100.0	17,558
Urban	38.2	61.8	100.0	12,869

Level of education ***				
Higher than secondary	23.1	76.9	100.0	1,246
Secondary level	35.1	64.9	100.0	2,192
Primary/elementary level	37.8	62.2	100.0	21,949
No schooling	23.1	76.9	100.0	5,040

Marital status ***				
Married	43.4	56.6	100.0	18050
Widowed/separated/divorced	20.5	79.5	100.0	11281
Single	35.4	64.6	100.0	1096

Total number of children ***				
None	37.4	62.6	100.0	1,750
1-2 children	40.1	59.9	100.0	6,991
3-4 children	37.9	62.1	100.0	10,683
5 or more children	27.4	72.6	100.0	11,003

Head of the household ***				
No	14.2	85.8	100.0	5,667
Yes	37.2	62.8	100.0	6,781

Living with children ***				
Not living with children	40.6	59.4	100.0	12,685
Living with children	30.3	69.7	100.0	17,742

Average total income per year ***				
100,000 Baht or more	39.6	60.4	100.0	4,867
30,000-99,999 Baht	45.7	54.3	100.0	10,148
10,000-29,999 Baht	29.8	70.2	100.0	10,391
Less than 10,000 Baht	17.1	82.9	100.0	4,995

Worked/working for the government/govt enterprise***				
No	35.7	64.3	100.0	28,010
Yes, government/Gov enterprise	21.3	78.7	100.0	2,417

Someone to assist incase financial problems **				
No	33.3	66.7	100.0	7,545
Yes	35.0	65.0	100.0	22,882

Currently in debt ***				
No	31.3	68.7	100.0	17,380
Yes, I am in debt	60.6	39.4	100.0	2,796
Yes, other persons are in debt	23.3	76.7	100.0	7,311
Yes, myself and other are in debt	57.3	42.7	100.0	2,940

Functional Status ***				
Able to all activities in daily living (ADL)	47.3	52.7	100.0	18,805
Difficulty in 1 and 2 ADL	21.8	78.2	100.0	5,522
Difficulty in 3 or more ADL	6.8	93.2	100.0	6,100

Number of chronic diseases ***				
No	41.1	58.9	100.0	17,073
1 disease	29.5	70.5	100.0	8,885
2 diseases	21.6	78.4	100.0	3,506
3 or more diseases	13.5	86.5	100.0	963

Number of psychosocial symptoms ***				
0	40.7	59.3	100.0	9,513
1-2	33.9	66.1	100.0	8,991
3-4	33.7	66.3	100.0	6,298
5 or more	26.3	73.7	100.0	5,625

Self assessed health status ***				
Good health	45.1	54.9	100.0	13,347
Poor health	26.4	73.6	100.0	17,080

**Total**	**34.6**	**65.4**	**100.0**	**30,427**

Note: *** = p < 0.001 **= p < 0.01 * = p < 0.5

Logistic regression analysis was used to assess the net effect of each of the independent variables on the dependent variable, while controlling for the other variables in the model. Three models were used in the analysis. The first model included the variable "sex of the respondent" and the effect of sex of the respondent on labor force participation was observed. The second model included other demographic and socioeconomic characteristics. In the third model, variables related to health behaviors were added and the effects of demographic, socioeconomic, and health behavior variables on labor force participation were observed.

The first model showed that the sex of the respondents was a significant predictor of labor force participation; females were 57% less likely (OR = 0.43) to be in the labor force than were males. In the second model, sex of the respondent retained its significance level even after inclusion of other demographic and socioeconomic characteristics. Analysis further found that age, place of residence, level of education, marital status, total number of children, household headship, living arrangements, average annual family income, employment in the government or in a government enterprise, and debt status were significant predictors for labor force participation. The analysis revealed that respondents aged 70-79 years and 80 years or above were 63 per cent (OR = 0.37) and 89 per cent (OR = 0.11), respectively, less likely to be working compared to those aged 60-69 years. Regarding the place of residence, urban respondents were more likely (OR = 1.25) to be in the labor force than were rural respondents. Similarly, those respondents who had only a primary level/elementary education or no schooling at all were about twice as likely to be in the labor force than were respondents with a secondary or higher education.

Widowed and single elderly were less likely to be in the labor force than were married respondents. Similarly, respondents who had more children were less likely to be working compared to those who did not have any children. Among those who had children, those who were living with their children were 31 per cent (OR = 0.69) less likely to be in the labor force than those who were not living with their children. Contrary to expectations, those whose family income was low were less likely to be in the labor force than those whose family income was high. Likewise, those who worked in the government sector were less likely to be in labor force as they got older than were those who had never worked for the government. Finally, respondents who were in debt were twice as likely to be in the labor force as were those who were not in debt.

All these variables retained their significance level even after inclusion of health behavior variables in the third model. Model 3 further illustrated that functional status, number of chronic diseases, and self-assessed health status were also important predictors of the likelihood of being in the labor force. Respondents who had more difficulty in daily living were less likely to participate in the labor force than were those who had no difficulty in daily living. For instance, respondents who had difficulty with 1 or 2 ADLs or difficulty with 3 or more ADLs were 43 per cent (OR = 0.57) and 81 per cent (OR = 0.19), respectively, less likely to be working than were those who had no such difficulty. Similarly, respondents who had 1, 2 or 3 or more chronic diseases were, respectively, 21 per cent (OR = 0.79), 43 per cent (OR = 0.57), and 52 per cent (OR = 0.48) less likely to report that they were in the labor force compared to those who had no chronic disease. Furthermore, respondents who assessed their health status as poor were 25 per cent less likely (OR = 0.75) to be in the labor force than were those who assessed their health status as good (Table 2).

**Table 2:** Adjusted odds ratio (OR) of reported having involvement in labor force participation in the past 7 days among Thai elderly by selected predictors

Predictors	Model I	Model II	Model III
**Demographic, socio-economic characteristics**			

**Sex of the respondent **Male (ref.)	1.00	1.00	1.00
Female	0.43***	0.53***	0.56***

**Age group **60-69 years (ref.)		1.00	1.00
70-79 years	-	0.37***	0.47***
80 years or +	-	0.11***	0.21***

**Place of residence **Rural (ref.)		1.00	1.00
Urban	-	1.25***	1.22***

**Level of education **Higher than secondary (ref.)		1.00	1.00
Secondary level	-	1.59**	1.79**
Primary/elementary level	-	2.01***	2.39***
No schooling	-	1.92***	2.45***

**Marital status **Married (ref.)		1.00	1.00
Widowed/separated/divorced	-	0.78**	0.85*
Single	-	0.91	1.02

**Total number of children **None (ref.)		1.00	1.00
1-2 children	-	0.64**	0.72*
3-4 children	-	0.55***	0.63**
5 or more children	-	0.49***	0.57**

**Head of the household **No (ref.)		1.00	1.00
Yes	-	2.06***	1.98***

**Living with children **Not living with children (ref.)		1.00	1.00
Living with children	-	0.69***	0.69***

**Average total income per year **100,000 Baht or more (ref.)		1.00	1.00
30,000-99,999 Baht	-	0.95	0.94
10,000-29,999 Baht	-	0.53***	0.54***
Less than 10,000 Baht	-	0.31***	0.35***

**Worked/working for the govt./govt. enterprise **No (ref.)		1.00	1.00
Yes, government/Gov enterprise	-	0.33***	0.33***

**Someone to assist incase financial problems **No (ref.)		1.00	1.00
Yes	-	0.99	0.91

**Currently in debt **No (ref.)		1.00	1.00
Yes, I am in debt	-	2.41***	2.28***
Yes, other persons are in debt	-	1.02	1.04
Yes, myself and other are in debt	-	2.12***	2.02***

**Health Behavior**			

**Functional status **Able to do all activities in daily living (ref.)			1.00
Difficulty in 1 and 2 ADL	-	-	0.57***
Difficulty in 3 or more ADL	-	-	0.19***

**Number of chronic diseases **No (ref.)			1.00
1 Disease	-	-	0.79***
2 diseases	-	-	0.57***
3 or more diseases	-	-	0.48***

**Number of psychosocial symptoms **0 (ref.)			1.00
1-2	-	-	1.06
3-4	-	-	1.06
5 or more	-	-	0.93

**Self assessed health status **Good health (ref.)			1.00
Poor health	-	-	0.75***

**Intercept**	0.83	0.93	1.16

**-2 Log likelihood**	33062.9	11665.6	10973.3

**Cox & Snell R Square**	0.038	0.200	0.243

Note: *** = p < 0.001 ** = p < 0.01 * = p < 0.5

## Discussion

This paper aims to examine the prevalence of labor force participation and to investigate the factors affecting labor force participation among those 60 years of age and above in Thailand. Sex of the respondents was a significant factor in determining labor force participation in all three models. All other demographic and socioeconomic variables, except for the variable "having someone to assist in case financial problems" in second model, were significant predictors of labor force participation. Even after including health behaviour variables in the third model, all previous variables retained their significance levels. Furthermore, among health behavior variables added in the model, all variables except psychosocial symptoms were significant. Older people, who were female, those who had more children, those living with children, those with less annual family income, those who had worked in government jobs, those with functional limitations, those with multiple chronic diseases, and those who perceived themselves to be in poor health were all associated with withdrawal from the labor force. On the contrary, those with less education, those who were heads of households and those indebt were quite likely to continue working in old age.

The present study found that labor force participation was higher among men than among women. This may be due to the expectations of women, regardless of their age, to stay home and take care of the household. Similarly, urban elderly exhibited a higher participation rate than did their rural counterparts. This could be due to the greater opportunities for employment in urban areas than in rural areas. While in most countries widowhood is associated with lower economic well-being, and, thus, countries with higher rates of widowhood would be expected to have higher labor force participation rates among older women [17], our study gave a different picture in this regard. Our study showed that widowed/separated/divorced older people were less likely to work than those who were married. This could be the case since in Thailand children are traditionally responsible for taking care of their parents in their old age. Those who had 5 or more children or those who lived with their children were less likely to work as they got older, which suggests that the children looking after their parents' livelihood. Knodel presents evidence to this effect of substantive intergenerational economic and emotional support from children toward their Thai parents [18]. Another reason could be that the government supports older people by distributing 500 Baht ($16) to them each month and that widowed/separated/divorced people are covered under this program.

Pressures from debts, responsibilities as head of household, and residence in a rural area may compel older people to continue working. But our findings contradict the human capital theory, which suggests that educational attainment is a person's human capital and that this is related to his or her labor force participation; the greater a person's human capital, the higher the probability of his/her participation in the workforce [19]. The presence in the labor force job market of older people with lower levels of education could be explained by the fact that the large agricultural sector allows for increased opportunities for such older, less educated people to continue working [17].

Ghosh notes that the health status of the aged plays a vital role in determining whether or not they participate in the labor force [20]. As people suffer from chronic diseases and different types of disabilities in old age their labor force participation is severely affected. However, their productivity increases when their health is improved [10]. Generally, participation in the labor force decreases with increases in age, and advanced age tends to limit the activities of daily living (eating, dressing, going to the toilet, bathing, sitting up, etc.) that one can perform [21]. Alaviniz and Burdorf found that in many European countries poor health, chronic diseases, and lifestyle factors are associated with being out of the labor market [14]. The present study found that age and multiple functional disabilities restrict old people's participation in the labor force. Chronic disease plays a detrimental role in old age, and if people are affected by multiple chronic diseases, the effect on their physical health is greater. A large number of older people in Thailand suffer from either a single illness or multiple chronic diseases, but many of them are still working. Although we could not examine information regarding their health service utilization or their life styles due to limitations of data, these factors might also contribute labor force participation.

There are some limitations in the interpretation of the results of this study. Because of the survey's cross-sectional design, all of the factors analyzed in the study were measured at a single point in time. Thus the analysis can only provide evidence of statistical association between those items and the labor force participation of elderly; it cannot show a cause-effect relationship. In addition, there may be other important factors such as coverage by an income security program, pensions, per capita income, the decision to participate in labor force, respondents' quality of life, etc. that affect labor force participation among the older population, but we were unable to examine the relationship of these variables to labor force participation in our study due to limitations in the data.

## Conclusion

Labor force participation among those over 60 years old is not uncommon in Thailand. Many factors determine the participation of old people in the labor force in Thailand. Among such factors, place of residence, functional status, and number of chronic diseases are the most significant predictors. The results suggest that improving the health status of the elderly is necessary in order to encourage employment among older persons. Thus, the country can address the labor shortage and further improve the economic condition of the nation. The results of this study also suggest that policies to encourage employment among older persons need to focus especially on the rural elderly.

## Competing interests

The authors declare that they have no competing interests.

## Authors' contributions

RA and KS conducted data analysis, interpreted the data, and drafted the manuscript. FH was involved in the interpretation of the results. All authors read and approved the final manuscript.

## Pre-publication history

The pre-publication history for this paper can be accessed here:

http://www.biomedcentral.com/1471-2318/11/15/prepub
